# Circulating and Tissue Expression Profile of MicroRNAs in Primary Hyperparathyroidism Caused by Sporadic Parathyroid Adenomas

**DOI:** 10.1002/jbm4.10431

**Published:** 2020-12-03

**Authors:** Maria P Yavropoulou, Kalliopi Pazaitou‐Panayiotou, John G Yovos, Christos Poulios, Athanasios D Anastasilakis, Dimitris Vlachodimitropoulos, Kyriakos Vambakidis, Olga Tsave, Sofia Chrisafi, Emily Daskalaki, Polyzois Makras

**Affiliations:** ^1^ Endocrinology Unit, 1st Department of Propaedeutic and Internal Medicine, Medical School National and Kapodistrian University of Athens Athens Greece; ^2^ Department of Medical Research 251 Hellenic Air Force & VA General Hospital Athens Greece; ^3^ Interbalkan Medical Center Thessaloniki Greece; ^4^ Faculty of Medicine Aristotle University of Thessaloniki Thessaloniki Greece; ^5^ Pathology Department, Faculty of Medicine Aristotle University of Thessaloniki Greece; ^6^ Department of Endocrinology 424 General Military Hospital Thessaloniki Greece; ^7^ Laboratory of Forensic Medicine and Toxicology, Medical School National and Kapodistrian University of Athens Athens Greece; ^8^ Department of Endocrine Surgery Henry Dunant Hospital Center Athens Greece

**Keywords:** MICRORNAs, ONCOGENES, PRIMARY HYPERPARATHYROIDISM, SPORADIC PARATHYROID ADENOMAS, TUMOR SUPPRESSOR GENES

## Abstract

We investigated the expression profile of selected microRNAs (miRs) in serum and tissue samples from patients with sporadic parathyroid adenomas (sPAs). This was a prospective, controlled cohort study. Forty patients with sPAs who had undergone parathyroidectomy (PTX) were included. MiR extraction was performed from (i) 40 formalin‐fixed paraffin‐embedded samples (FFPEs) of sPAs, (ii) 10 FFPEs of normal parathyroid tissue (NPT), (iii) serum samples of the 40 patients with sPAs (t1 = baseline; t2 = 2 months post‐PTX), and (vi) serum samples of 10 healthy individuals (controls; t1 = baseline and t2 = 2 months later). Ten miRs were selected based on their interaction with genes related to parathyroid tumorigenesis (miR‐17‐5p, miR‐24‐3p, miR‐29b‐3p, miR‐31‐5p, miR‐135b‐5p, miR‐186‐5p, miR‐195‐5p, miR‐330‐3p, miR‐483‐3p, and miR‐877‐5p). At tissue level, the relative expression of miR‐17‐5p, miR‐31‐5p, miR‐135b‐5p, miR‐186‐5p, and miR‐330‐3p was significantly decreased (fold change [FC]: 0.17, FC: 0.03, FC: 0.01, FC: 0.10, FC: 0.10, respectively; all *p* values <0.001), and the expression of miR‐24‐3p and miR‐29b‐3p was significantly increased (FC: 12.4, *p* < 0.001; FC: 18.5, *p* = 0.011, respectively) in sPA compared with NPT samples. The relative expression of miR‐135b‐5p was also significantly decreased in the serum samples of patients compared with controls (FC: 0.7, *p* = 0.035). No significant differences were found in the serum samples of patients before and after PTX. MiRs that regulate genes linked to parathyroid tumors such as menin 1 (miR‐24‐3p, miR‐29b‐3p), cyclin D1 (miR‐17‐5p), calcium sensing receptor (miR‐31‐5p, miR‐135b‐5p), cyclin‐dependent kinase inhibitors (miR‐186‐5p), and β‐catenin (miR‐330‐3p) were significantly deregulated in sPAs compared with NPT samples, suggesting a role for epigenetic changes in parathyroid tumorigenesis. © 2020 The Authors. *JBMR Plus* published by Wiley Periodicals LLC on behalf of American Society for Bone and Mineral Research.

## Introduction

Primary hyperparathyroidism (PHP) is one of the most common endocrinopathies characterized by hypercalcemia caused by increased secretion or inappropriate secretion of PTH from the parathyroid glands.^(^ ^1^ ^)^ Most cases (85%) are attributed to the development of a benign adenoma of clonal (monoclonal or oligoclonal) origin,^(^ ^1^ ^)^ which occurs sporadically at approximately 90% to 95% of cases. PHP occurring in the context of genetic familial syndromes, is mainly caused by multiglandular disease and is characterized by an earlier age of onset.^(^ ^2^, ^3^ ^)^

Several reports have implicated genes that are responsible for the heritable forms of PHP in the pathogenesis of apparently sporadic parathyroid adenomas (sPAs), either through somatic mutations or via predisposing germline mutations.^(^ ^4^ ^)^ Among them, the *MEN1* gene, encoding for menin protein, a tumor suppressor protein responsible for multiple endocrine neoplasia type 1 (MEN1) syndrome, and *CCND1* oncogene, encoding for cyclin D1 have been solidly established as primary tumorigenic drivers in sPAs.^(^ ^4^ ^)^ Other somatic mutations linked to sPAs include cyclin‐dependent kinase inhibitor 1B (*CDKN1B*), which encodes for P27KIP1 (p27) cell‐cycle regulator, responsible for MEN4 syndrome; calcium‐sensing receptor (*CaSR*) gene associated with familial hypocalciuric hypercalcemia and neonatal severe hyperparathyroidism; and *CTNNB1*, the gene encoding for beta‐catenin cytoplasmic protein, the transcriptional activator of canonical Wnt signaling.^(^ ^4^ ^)^

However, in the majority of sPAs, somatic mutations are not identified, suggesting that other molecular mechanisms are the initial drivers of parathyroid tumorigenesis. In recent years, the role of epigenetics has emerged in an attempt to explain significant differences in phenotypes among patients with the same disease.^(^ ^5^ ^)^ Epigenetic mechanisms influence gene expression in postnatal life without altering the DNA sequence. These mechanisms include DNA methylation, posttranslational modifications of histones, and posttranscriptional regulation of genes by noncoding RNAs. MicroRNAs (miRs) are small noncoding RNAs of 22 nucleotides in length that regulate the expression of target genes through complementary binding with the messenger RNA (mRNA) of the target gene and the formation of the miR‐induced silencing complex (miRISC).^(^ ^6^ ^)^ Binding of miRISC to the specific untranslated region (UTR) of mRNA molecules results in posttranscriptional gene repression induced by inhibition of translation or destabilization of mRNA.^(^ ^7^ ^)^ The magnitude of the translational repression of the target mRNA depends on the mRNA‐miR complementarity. In general, miRs are negative regulators of gene expression, although activation of the target mRNA translation has also been reported.^(^ ^8^ ^)^ Several studies have shown the role of miRs in the regulation of PTH synthesis and secretion, *CaSR* expression, and parathyroid cell tumorigenesis.^(^ ^9^, ^10^, ^11^, ^12^ ^)^ Recent studies have also identified the expression of certain miRs in MEN1 parathyroid neoplasia.^(^ ^13^, ^14^ ^)^ Mature miRs are released from the cell cytoplasm into the blood stream via extracellular vesicles^(^ ^15^ ^)^; a growing body of evidence has shown that tissue expression of miRs is highly associated with their expression in the serum.^(^ ^16^, ^17^, ^18^, ^19^ ^)^ Currently, data are scarce regarding the expression profile of circulating miRs in the serum of patients with PHP caused by sPAs. To address this issue, we investigated the tissue and serum expression profile of miRs that regulate the expression of genes linked to the development of sPAs in a cohort of patients with nonfamilial PHP.

## Patients and Methods

### Study design

This was a multicenter, prospective, controlled cohort study. Adult patients with biochemical diagnosis of PHP and identification of a PA in imaging techniques who were subjected to parathyroidectomy (PTX) were enrolled. The inclusion criterion was the localization of a single abnormal parathyroid gland. Patients with (i) a family history of PHP, (ii) a personal/family history suggestive of multiple endocrine neoplasia or related syndromes, (iii) evidence of atypical or malignant features in the histology report, (iv) parathyroid carcinomas according to Schantz and Castleman's histologic criteria,^(^ ^20^ ^)^ (v) parathyroid hyperplasia or more than one PA, (vi) chronic kidney disease stage 3 and above, (vii) liver failure, (viii) any type of cancer, and (ix) medication known to affect calcium metabolism (eg, glucocorticoids, calcimimetics, bisphosphonates, and denosumab) were excluded from further analysis.

Parathyroid tissue samples along with histopathological data and blood samples at two time points (baseline and 2 months postsurgery) were collected from the enrolled patients. Normal parathyroid tissue (NPT) samples obtained accidentally at the time of total thyroidectomy for benign multinodular goiter served as tissue controls. Blood samples from other healthy individuals with normal calcium metabolism at two time points (t1 = baseline and t2 = 2 months later without any intervention) were used as serum controls.

Forty patients with PHP (5 males and 35 females) caused by sPAs were included in the study. Additionally, NPT samples from 10 individuals (1 male and 9 females) who underwent total thyroidectomy (tissue controls) and blood samples from 10 other healthy individuals (1 male and 9 females; serum controls) were analyzed. All 20 individuals serving as controls with either their normal parathyroid tissue sample (tissue controls) or their blood samples (serum controls) were age‐matched with the patients with PHP, had normal calcium levels and lack any of the exclusion criteria.

All patients and controls signed an informed consent form, and the study was approved by the scientific committees of AHEPA University Hospital and 251 Hellenic Air Force and VA General Hospital.

### Histopathological and immunohistochemical analysis

Tissue samples from all enrolled patients and tissue controls were collected, fixed in formaldehyde 10%, and embedded in FFPE blocks. From each block, five sections (10 μm) were used for RNA extraction and one section (3 μm) was stained with H&E for morphological evaluation and verification of PA or NPT. In addition, the H&E slides were used for marking the epithelial‐rich areas for microdissection and tumor microarray (TMA) construction as previously described.^(^ ^21^ ^)^ From each block, three 1‐mm‐wide cores were taken to include sufficient tissue area. From each TMA block, five slides (3 μm) were produced, one was stained with H&E and the rest were used for immunohistochemical evaluation. We performed an immunohistochemical evaluation of adenomatous polyposis coli (APC; C‐20 clone; Santa Cruz Biotechnology, Santa Cruz, CA, USA), Ki67 (MIB1 clone; DAKO, Carpinteria, CA, USA), cyclinD1 (EP12 clone; DAKO), and parafibromin (2H1 clone; Santa Cruz Biotechnology) expression to definitely establish a diagnosis of PA and exclude cases of atypical PA and/or parathyroid carcinoma.^(^ ^22^, ^23^, ^24^ ^)^ All immunohistochemical stains were performed using the Bond Max and Bond III autostainers (Leica Microsystems, Wezlar, Germany). Parafibromin was evaluated as “positive” for any percentage of nuclear positivity and “negative” for no nuclear positivity. For APC, cytoplasmic positivity was evaluated as a percentage of positive cells. For cyclinD1 and Ki67, nuclear positivity was evaluated as a percentage of positive cells. Each stain was evaluated in three cores, and the average value was taken into account for statistical analysis.

#### RNA extraction from paraffin‐embedded tissue

Manual microdissection was performed from each FFPE block to enrich for epithelial tissue before total RNA extraction as previously described.^(^ ^21^ ^)^ As mentioned above, the target lesion was marked on the H&E slide for each case, which was then used to guide the removal of nontarget tissues (eg, normal parathyroid tissue) from unstained slides. After an overnight deparaffinization, the tissue area of interest was scraped off the slide into a microcentrifuge tube to facilitate total RNA extraction. MicroRNA extraction was performed by miRNeasy FFPE kit (QIAGEN Inc, Valencia, CA, USA), according to the manufacturer's instructions. The kit is optimized to isolate RNA molecules longer than 18 nucleotides from FFPE tissue samples, and to reverse as much formaldehyde modification as possible without further RNA degradation. Total RNA was extracted using the commercial RNeasy FFPE kit (QIAGEN GmbH, Hilden, Germany), according to the manufacturer's instructions.

The concentrations of the RNA samples were determined by OD260 using a NanoDrop ND‐1000 instrument (Thermo Fisher Scientific, Massachusetts, USA). The integrity of RNA was assessed by electrophoresis on a denaturing agarose gel.

#### Quantitative real‐time reverse transcription polymerase chain reaction

Following RNA extraction from the FFPE samples of both sPA and NPT cDNA was synthesized with the miScript II RT kit (QIAGEN) for miR analysis and the RT^2^ First Strand kit (QIAGEN, GmbH), for mRNA analysis according to the manufacturer's instructions. Samples were kept at −20^°^C until the subsequent qRT‐PCR analysis. Cycling was performed under standardized conditions with (i) the 2x QuantiTect SYBR Green PCR Master Mix for the miR‐specific primers (Supplementary Table S1), and (ii) the Rotor‐Gene SYBR Green PCR kit for the relative expression of *MEN1* gene (Homo sapiens menin 1 transcript variant 1, linear mRNA, 2785 bp. NM_000244, RT^2^ qPCR Primer Assay; QIAGEN Inc) on the QIAGEN Rotor‐Gene Q (Corbett Rotor‐Gene 6000) RT‐PCR cycler. Relative miRNA and mRNA expression was calculated with the 2−ΔΔCT method. The relative expression levels of the *MEN 1* gene was determined by the cycle number via qRT‐PCR, normalized to the glyceraldehyde 3‐phosphate dehydrogenase (GADPH) cycle number using the 2DDCT method.

#### Selection of microRNA primer assays

Selection of miRs was based on existing literature on specific miRs that were reported to associate with genes linked to the pathogenesis of sPAs. In addition, the following databases: (i) miRbase,^17^ (ii) DIANA TOOLS,^18^ (iii) PicTar,^19^ (iv) miRDB,^20^ (v) TargetScanHuman,^21^ (vi) miRGator, ^22^ and (vii) microRNA^23^ were searched to identify biological targets of the selected miRs in humans, searching for 8mer, 7mer, and 6mer sites that match the seed region for each miR, using conserved sites and the best cumulative scores. Ten miRs that fulfilled the above criteria were finally selected for analysis (Supplementary Table S1).

### Blood sampling

Following an overnight fast, morning blood samples were obtained from patients at two time points: t1 = baseline and t2 = 2 months post‐surgery. Blood samples from the 10 healthy individuals that served as serum controls were also obtained at two time points: t1 = baseline and t2 = 2 months later (without any intervention). Serum was separated and stored at −80°C until further analysis. A total of 200 μL from each sample was used for purification of cell‐free total RNA: primarily miRNA by miRNeasy Serum/ Plasma kit (QIAGEN GmbH), according to the manufacturer's instructions. Reverse transcription was performed with the miScript II RT kit (QIAGEN, GmbH). Cycling was performed under standardized conditions with 2x QuantiTect SYBR Green PCR Master Mix on the QIAGEN Rotor‐Gene Q (Corbett Rotor‐Gene 6000) RT‐PCR cycler. Performance of qRT‐PCR was done in triplicates.

#### Quality control

To determine the efficiency of RNA extractions from serum and/or the presence of inhibitors in cDNA synthesis or in qRT‐PCR, we quantified the levels of the spike‐in control (*Caenorhabditis elegans* miR‐39 miR mimic) added before RNA extraction by qRT‐PCR. All cDNAs expressed the spike‐in control at normal levels (cycle threshold [CT] <22 cycles). Statistical analysis confirmed the absence of outliers in the CT values for the spike‐in.

#### Biochemical assays

Fasting blood samples at each time point from serum controls and patients with PHP were collected for the measurement of serum creatinine, serum calcium, serum phosphorus, serum albumin, and 24‐hour urine calcium using standard laboratory methods. Serum PTH and serum 25‐OH‐vitamin D levels were measured by a second‐generation electrochemiluminescence immunoassay on Cobas e411 automated analyzer (Roche Diagnostics GmbH, Mannheim, Germany) according to the manufacturer's instructions. The measurement range for intact PTH was 1.2 to 5000 pg/mL and the within‐run and total run assay coefficients of variation were between 0.9% and 2.8% and 1.6% and 3.4%, respectively. The measurement range for total 25(OH) D levels was 3 to 100 ng/mL and the within‐run and total run assay coefficients of variation were <10.5% and <4%, respectively.

### Statistical analysis

Resultant data on mean CT values for each miR were exported and uploaded to the QIAGEN website for analysis (miR primer assay data analysis version 3.5, GeneGlobe Data Analysis), where a classic ΔΔCT calculation and a log2 transformation provided normalized fold‐difference values for the miR targets as previously described.^12^ The *p* values were calculated based on (i) Student's paired *t* test of the replicate 2̂ (−ΔΔCT) values for each miR between the different time points of blood samples within the group of patients or serum controls, and (ii) Student's independent *t* test of the replicate 2̂ (−ΔΔCT) values for each miR in blood samples between patients and serum controls and in tissue samples between sPAs and NPT. Data on biochemical and anthropometric results are given as mean ± SD.

Normal distribution was evaluated by the Kolmogorow‐Smirnov test. One‐way ANOVA followed by Bonferroni's multiple comparison tests and/or Dunnett test or a Kruskal‐Wallis test was performed to assess differences between groups, as applicable. Pearson correlation coefficient or Spearman's rank correlation coefficient was used for associations between relative serum and tissue expression of the selected miRs and calcium metabolism and histochemical characteristics of sPAs in the patient group, as applicable.

All *p* values are two‐sided and a value of <0.05 was considered as statistically significant. All statistical analyses were performed using the Statistical Package for the Social Sciences (SPSS, Chicago, IL, USA) version 23.0.

## Results

### Patient characteristics

Demographic characteristics and biochemical profile of all participants are given in Table 1. Mean age of the patients with PHP was 57.5 years ±10.7. All patients had normalized serum calcium and PTH levels following PTX (Table 1).

**Table 1:** Αnthropometric and Laboratory Values of the Study Participants

Parameters	Patients with PHP *n* = 40		Serum controls (serum samples) *n* = 10	Tissue controls (tissue samples) *n* = 10
Age (y)	57.5 ± 10.7		55.2 ± 10.5^a^	54.2 ± 8.2^a^
Sex (male/female)	5/35		1/9^a^	1/9^a^
Age at menopause (y)	49.2 ± 4.4		48.5 ± 5.2^a^	50.1 ± 2.1^a^

Biochemical profile	Baseline	2 m post‐PTX	Baseline	Examinations performed before total thyroidectomy^b^
sCa^c^ (mg/dL) (NR: 8.2–10.2)	11.2 ± 2.4^d^ ^,^ ^e^	8.8 ± 1.1	9.2 ± 1.3	9.0 ± 1.1
sPO_4_ (mg/dL) NR: 2.5–4.5	2. 8 ± 0.9^d^ ^,^ ^e^	3.1 ± 0.5	3.0 ± 0.09	3.4 ± 0.7
sCreatinin (mg/dL) NR: 0.9–1.3	1.1 ± 0.3^d^ ^,^ ^e^	0.92 ± 0.08	0.89 ± 0.06	0.77 ± 0.9
sPTH (pg/mL) NR: 10–65	87 ± 10.9^d^ ^,^ ^e^	55 ± 7.1	49 ± 4.5	45 ± 5.9
s25‐OH‐vitamin D (ng/mL)	17.6 ± 6.8	‐	18.8 ± 4.5	NA
24‐h Urine Ca (mg/24 h) NR:100–300	285 ± 42 .4^d^ ^,^ ^e^	134 ± 22.8	128 ± 20.5	NA

Ca = calcium; NA = Not available; NR = normal range; PHP = primary hyperparathyroidism; PTX = parathyroidectomy; s = serum.

^a^ Nonsignificant: comparisons performed between the group of patients and serum or tissue controls.

^b^ Blood samples were not available for tissue controls, but we retrieved the biochemical tests for calcium metabolism of the enrolled participants from the hospital's medical database.

^c^ sCa levels are corrected to serum albumin levels.

^d^ *p* < 0.05 compared with serum samples from controls at baseline (t1).

^e^ *p* < 0.05 compared with serum samples at 2 months post‐PTX.

### Immunohistochemical findings

All tested samples, both sPAs and NPT were positive for parafibromin (Supplementary Table S2). APC expression, based on the percentage of “positive” cells in the cytoplasm, showed no significant differences between the two groups, though it was slightly higher in the sPA compared with the NPT group (average percentage of “positive” cells in NPT group = 48% versus sPAs group = 60%). Ki67 expression was low in all cases of sPA, reaching up to 1% and negative in all NPT samples. Nuclear expression of cyclinD1 was significantly higher in sPAs compared with NPT samples, as previously described.^(^ ^21^ ^)^ In particular, positive nuclear staining was found in 10% of NPT and 95% of sPA sanples (Supplementary Table S2).

### Differential miR expression profile between sporadic parathyroid adenomas and normal parathyroid tissue

**Fig 1:**
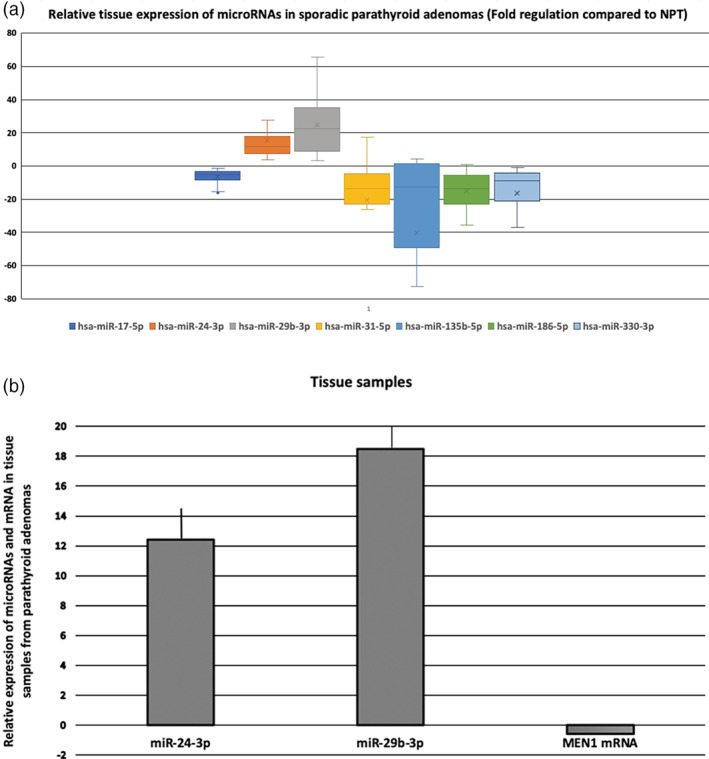
(*A*) Boxplot with the relative tissue expression of the tested miRs in tissue samples from sporadic parathyroid adenomas compared with tissue samples from normal parathyroid tissue. Values are expressed as gene fold regulation compared with normal parathyroid tissue and are displayed in a standardized way (*n* = 38; minimum = 25th percentile; median = 75th percentile; maximum and mean values [x]). (*B*) Relative tissue expression of miR‐24‐3p, miR‐29b‐3p, and MEN1 mRNA in sporadic parathyroid adenomas (*n* = 38). Values are expressed as gene fold changes compared with normal parathyroid tissue. hsa = Homo‐sapiens; miR = microRNA; mRNA = messenger RNA; NPT = normal parathyroid tissue.

At tissue level, the miR expression profile differed significantly between the sPA and NPT groups (Table 2; Fig. 1*A*). Specifically, the relative expression of 5 out of the 10 miRs tested, namely miR‐17‐5p, miR‐31‐5p, miR‐135b‐5p, miR‐186‐5p, and miR‐330‐3p, was decreased (all *p* values <0.001) in the sPA group compared with the NPT group (Table 2). Yet the relative expression of two miRs that target the *MEN1* gene was increased in the sPA group (miR‐24‐3p, fold change [FC]: 15.6, *p* < 0.001; miR‐29b‐3p, FC: 24.8, *p* = 0.011), compared with the NPT group. No correlation was found between the differential expression of the prespecified panel of miRs in sPAs and the histochemical characteristics of the parathyroid tumors (ie, size of the tumor and Ki67 proliferation marker). The relative tissue expression of miR‐17‐5p was negatively correlated with the immunohistochemical expression of cyclin D1 in sPAs (*r* = − 0.543, *p* = 0.032).

**Table 2:** MicroRNA Expression Profile in Parathyroid Adenomas Compared With Normal Parathyroid Tissue

MicroRNAs	Fold change	*p* Value	Predicted gene	Predicted mechanism of action
hsa‐miR‐17‐5p	0.17	<0.001	Cyclin D1 (*CCND1*)	Upregulation of the oncogene *CCND1* Favors tumorigenesis
hsa‐miR‐24‐3p	15.6	<0.001	Menin (*MEN1*)	Downregulation of the tumor suppressor gene MEN1 Favors tumorigenesis
hsa‐miR‐29b‐3p	24.8	0.011	Menin (*MEN1*)	Downregulation of the tumor suppressor gene *MEN1* Favors tumorigenesis
hsa‐miR‐31‐5p	0.03	<0.001	Calcium‐sensing receptor (*CASR*)	Upregulation of the tumor suppressor gene *CASR* Suppresses tumorigenesis
hsa‐miR‐135b‐5p	0.01	<0.001	Calcium‐sensing receptor (*CASR*)	Upregulation of the tumor suppressor gene *CASR* Suppresses tumorigenesis
hsa‐miR‐186‐5p	0.10	<0.001	Cyclin‐dependent kinase inhibitor 1B (*CDKN1B*)	Upregulation of the tumor suppressor gene *CDKN1B* Suppresses tumorigenesis
hsa‐miR‐195‐3p	1	0.367	Cyclin D1 (*CCND1*)	
hsa‐miR‐330‐3p	0.10	<0.001	Catenin‐beta (*CTNNB1*)	Upregulation of the oncogene *CTNNB1* Favors tumorigenesis
hsa‐miR‐483‐3p	Undetectable^a^	—	Catenin‐beta (*CTNNB1*)	
hsa‐miR‐877‐5p	Undetectable^a^	—	Cyclin‐dependent kinase inhibitor 1B (*CDKN1B*)	

Hsa = Homo‐sapiens; miR = microRNA.

^a^ This miRNA's average molecular tag count, and thus its expression level, was undetectable in either the control (normal parathyroid tissue) or the test group (parathyroid adenomas); thus no further analysis could be performed.

We next examined the relative expression of MEN1 mRNA in the tissue samples from patients with PHP compared with controls. In agreement with the tissue relative expression of miR‐24‐3p and miR‐29b‐3p, the relative expression of MEN1 mRNA was significantly decreased in sPAs compared with NPT (FC: 0.58, *p* = 0.043; Fig. 1*B*).

### Differential miR expression profile in the serum of patients with sporadic parathyroid adenomas and controls

In serum samples, only the relative expression of miR‐135b‐5p was found decreased in patients compared with serum controls (FC: 0.7, *p* = 0.035; Table 3).

**Table 3:** MicroRNA Expression Profile in the Serum of Patients With PHP at Baseline and After Parathyroidectomy Compared With Serum Controls

MicroRNAs	Patients with PHP			
	Baseline		2 months post‐PTX	
	Fold change	*p* Value^a^	Fold change	*p* Value^a^
hsa‐miR‐17‐5p	1.60	0.633	1.30	0.225
hsa‐miR‐24‐3p	>100	0.659	>100	0.080
hsa‐miR‐29b‐3p	1.50	0.657	8.40	0.331
hsa‐miR‐31‐5p	Undetectable^b^	—	Undetectable^b^	—
hsa‐miR‐135b‐5p	0.70	0.035	0.80	0.048
hsa‐miR‐186‐5p	2.50	0.660	2.70	0.218
hsa‐miR‐195‐3p	Undetectable^b^	—	Undetectable^b^	—
hsa‐miR‐330‐3p	1.00	0.930	1.60	0.300
hsa‐miR‐483‐3p	0.08	0.747	0.18	0.231
hsa‐miR‐877‐5p	14.14	0.623	3.2	0.801

Hsa = homo sapiens; miR = microRNA; PHP = primary hyperparathyroidism; PTX = parathyroidectomy.

^a^ Comparisons are performed with baseline serum samples of controls.

^b^ This miRNA's average molecular tag count, and thus its expression level, was undetectable in either the control (normocalcemic serum controls) or the test group (patients with PHP at baseline and 2 months post‐PTX); thus no further analysis could be performed.

We next tested whether PTX and the subsequent normalization of calcium and PTH levels (Table 1) would affect the expression profile of the prespecified panel of miRs. Interestingly, the relative expression of all tested miRs was invariable between t1 and t2 in the patient group (Table 3). As expected, no differences over time were found in the serum samples from controls (data not shown).

In serum samples at 2 months post‐PTX, a marginal decrease of the relative expression of miR‐135b‐5p was also observed in patients compared with controls (Table 3).

In patients with PHP, no correlations were identified between baseline serum expression profile of miRs and serum levels of PTH or other calcium metabolism parameters.

## Discussion

In the present study, we report significant differences in the tissue miRs expression profile of sPA compared with NPT. In particular, we found decreased relative expression of miRs that regulate *CaSR* (miR‐31‐5p and miR‐135‐5p), *CDKN1B* (miR‐186‐5p), *CTNNB1* (miR‐330‐3p), and *CCND1* (miR‐17‐5p) genes; and increased expression of miRs that regulate the *MEN1* gene (miR‐24‐3p and miR‐29b‐3p) in sPAcompared with NPT (Fig. 1). In line with the tissue miR expression pattern, we also found decreased expression of miR‐135b‐5p in the serum of patients with PHP compared with normocalcemic controls, both at baseline and after PTX, despite the normalization of calcium and PTH levels.

**Fig 2:**
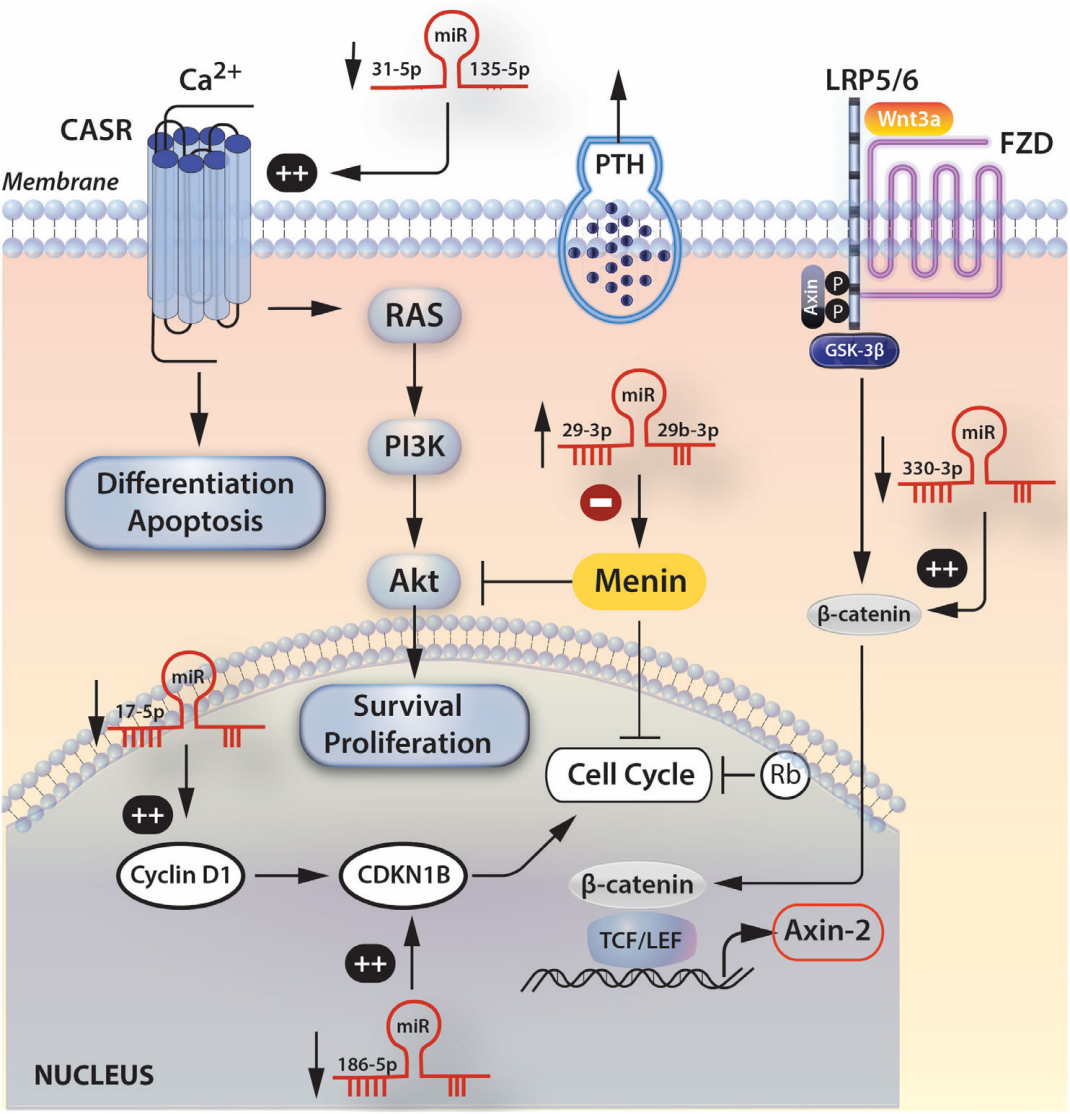
Proposed model of epigenetic regulation of parathyroid tumorigenesis based on the deregulated expression profile of miRs and their predicted genes in sporadic parathyroid adenomas. **++** = Predicted increased gene expression via the interaction with the relative miRs; ‐ = predicted decreased gene expression via the interaction with the relative miRs; ↑ = increased relative expression of the indicated miRs. ↓ = decreased relative expression of the indicated miRs. β‐cat = beta catenin; Akt = protein kinase B; APC = adenomatous polyposis coli; Ca2+ = extracellular calcium; CaSR = calcium sensing receptor; CDKN1B = cyclin‐dependent kinase 1B; FZD = Frizzled; GSK = glycogen synthase kinase; LEF = lymphoid enhancer factor; LRP = Low‐density lipoprotein receptor‐related protein; miR = microRNA; PI3K = phosphoinositide 3‐kinase; PTH = parathyroid hormone; Ras = small momomeric GTP‐binding protein; TCF = T‐cell factor.

Differential tissue miR expression patterns have been reported in parathyroid carcinomas compared with NPT,^(^ ^9^, ^10^, ^25^ ^)^ but data on sPAs are scarce. Parathyroid carcinomas show a global downregulation (approximately 80%) of miRs compared with NPT,^(^ ^9^, ^25^ ^)^ reflecting the general cancer‐associated behavior of miRs repression. Corbetta and colleagues^(9)^ identified four miRs, namely miR‐296, miR‐139, miR‐222, and miR‐503, implicated in the control of cell‐cycle progression genes, as being significantly deregulated in parathyroid carcinoma compared with NPT.^(^ ^9^ ^)^ Aberrant expression of miR‐222 in parathyroid carcinoma was also shown by Rahbari and colleagues.^(^ ^25^ ^)^ In addition, expression of the C19MC cluster of miRs, an important driver of tumorigenesis and metastasis,^(^ ^26^, ^27^ ^)^ was significantly enriched in parathyroid carcinomas and distinguished carcinomas from PAs.^(^ ^10^ ^)^ Several of these studies have also focused on identifying molecular biomarkers that can distinguish PAs from parathyroid carcinomas. MiR‐222^(^ ^28^ ^)^ and miR‐503 were downregulated in sPAs compared with parathyroid carcinomas.^(^ ^9^ ^)^ Conversely, miR‐296 was upregulated in sPAs compared with parathyroid carcinomas, whereas miR‐139 expression was similarly downregulated and miR‐517c similarly upregulated in both parathyroid tumors,^(^ ^29^ ^)^ supporting the role of a miR expression profile in distinguishing PAs from parathyroid carcinomas.

Studies evaluating the miR profile in MEN1‐linked parathyroid hyperplasia found increased expression of miR‐24 predicted to bind mainly the 3‐UTR of *MEN1* mRNA.^(^ ^13^, ^14^ ^)^ In particular, MEN1‐related parathyroid tumors harboring the *MEN1* loss of heterozygosity (LOH) at 11p13 (thus completely missing the expression of wild type MEN1 mRNA and menin protein) showed higher expression of miR‐24 compared with MEN1 PAs without LOH at 11q13 or somatic mutations of the *MEN1* gene. Moreover, expression of miR‐24 was also higher in MEN1‐related parathyroid tumors harboring the *MEN1* LOH at 11p13 compared with sPAs and normal parathyroid glands.^(^ ^13^ ^)^ In our patient cohort, we also found increased tissue expression of miR‐24‐3p and miR‐29b‐3p both targeting the *MEN1* gene, suggesting a role for the epigenetic repression of the *MEN1* gene in parathyroid tumorigenesis independent of the presence of a familial syndrome. Interestingly, we have shown that miR‐induced regulation of other oncogenes such as *CCND1* and *CTNNB1* may also contribute to the pathogenesis of sPAs. In addition, we showed increased cyclin D1 protein expression in sPAs compared with NPT, in line with what has been previously described^(^ ^21^, ^28^, ^30^ ^)^ which was associated with decreased relative expression of miR‐17‐5p (negative regulator of the *CCND1* gene).

In accordance with other studies that have reported strong associations between tissue and serum miRs expression in several diseases,^(^ ^31^, ^32^ ^)^ we found that the relative expression of miR‐135b‐5p was downregulated in both the tissue and serum of our patients with PHP. This finding would appear to be in conflict with the oncogenic role of this specific miR that has been consistently reported to be overexpressed and to downregulate the *CaSR* gene in various tumors.^(^ ^33^, ^34^ ^)^ However, it seems that the tissue expression of miRs in sPAs follows an expression pattern that can promote tumorigenesis, and at the same time exert an autoregulatory tumor suppression mechanism. In particular, the deregulated tissue expression of miR‐24‐3p, miR‐29b‐3p, miR‐17‐5p, and miR330‐3p favors parathyroid tumorigenesis, whereas the decreased relative expression of miRs that target the tumor suppressor genes *CaSR* (miRs −31‐5p and miR‐135b‐5p) and *CCND1* (miR‐186‐5p) suppresses tumorigenesis (Fig. 2).

Surprisingly, we did not find differences in the serum miR expression profile between baseline and post‐PTX serum samples, as we would expect because of the successful excision of the parathyroid tumor. However, because circulating miRs show remarkable stability in body fluids,^(^ ^32^ ^)^ we could speculate that a 2‐month follow‐up period was not enough for the clearance of the released miRs from the bloodstream. Another hypothesis would be that the deregulated expression pattern of miRs seen in sPAs reflects an intrinsic impaired regulatory mechanism of the parathyroid gland that allows the development of clonal PAs. This hypothesis could potentially explain the rarely observed development of PAs among patients with a history of surgically excised PAs or even cases of double PAs. However, because we did not have access to NPT in close proximity to the sPAs of our cohort, this hypothesis remains to be tested.

Our study has several limitations. First, we do not have data regarding potential somatic mutations in our samples, but because the contribution of somatic gene mutations in sporadic parathyroid disease accounts for <5% of cases,^(^ ^4^ ^)^ we feel that this would not significantly affect our results. Second, we do not have longer‐term follow‐up of post‐PTX to address more accurately the parathyroid disease‐free period. Third, we are aware that regarding the controls, both tissue (NPT) and serum samples should ideally be obtained from the same individuals. However, NPT can only be accidentally excised from individuals without parathyroid disease; therefore, no serum samples could have been obtained from these individuals. This is the reason for using a separate control group solely for serum comparisons. Fourth, we observed an heterogeny of gene expression for some of the tested miRs in the normal parathyroid tissue samples: specifically miR‐877, miR‐31, miR‐135b‐5p, miR‐186, and miR‐17‐5p. Finally, we did not have access to the rim of normal parathyroid tissue in close proximity to the removed PA, but the use of NPT of individuals without parathyroid pathology is a generally accepted method.

In conclusion—and to the best of our knowledge—this is the first study investigating the expression pattern of miRs related to specific oncogenes and tumor suppressor genes traditionally linked to PAs in both tissue and serum samples of patients with PHP. Our study implies a role of epigenetic deregulation in parathyroid tumors. Whether these epigenetic alterations could have a primary tumorigenic role or act synergistically with an impaired genetic background remains to be elucidated. Nevertheless, further research for specific miR molecular signatures in sPAs is highly needed: It can provide new approaches towards the development of novel diagnostic biomarkers and therapeutic molecular targets.

## Disclosure

The authors declare that there is no conflict of interest that could be perceived as prejudicing the impartiality of the research reported.

## Author Contributions

**Maria Yavropoulou:** Conceptualization; data curation; formal analysis; investigation; methodology; project administration; supervision; validation; writing‐original draft. **Kalliopi Pazaitou‐Panayiotou:** Funding acquisition; investigation; writing‐review and editing. **John Yovos:** Conceptualization; investigation; writing‐review and editing. **Christos Poulios:** Investigation; methodology; validation; writing‐review and editing. **Athanasios Anastasilakis:** Investigation; writing‐review and editing. **Dimitris Vlachodimitropoulos:** Methodology; resources; writing‐review and editing. **Kyriakos Vamvakidis:** Resources; validation. **Olga Tsave:** Formal analysis; investigation; methodology. **Sofia Chrisafi:** Investigation; methodology. **Emily Daskalaki:** Investigation; methodology. **Polyzois Makras:** Data curation; funding acquisition; project administration; supervision; validation; writing‐review and editing.

### Peer Review

The peer review history for this article is available at https://publons.com/publon/10.1002/jbm4.10431.

## Supporting information


**Supplementary Table S1** Pre‐specified panel of selected microRNAs\
