# Supplementary material for: Circulating and Tissue Expression Profile of MicroRNAs in Primary Hyperparathyroidism Caused by Sporadic Parathyroid Adenomas
Source: JBMR Plus. 2020 Dec 3;5(2):e10431. doi: 10.1002/jbm4.10431 (PMC7872342; doi:10.1002/jbm4.10431)
Supplement: Supplementary file 1 — Supplementary Table S1 Pre‐specified panel of selected microRNAs\ [file JBM4-5-e10431-s001.docx]

| Suppl. Table 1. Pre-specified panel of selected microRNAs | | | | |
| --- | --- | --- | --- | --- |
| Target Genes | **Mechanism of Action** | **MicroRNA**  **(validated) with Score >.85*** | MiScript Primer Assay  **(Catalog #,Qiagen)**  **Descritpion** | **MicroRNA sequence** |
| Cyclin D1 (CCND1/PRAD1) | **Oncogene** | **hsa-miR-17-5p** | MS00029274  Mature microRNA | 5'CAAAGUGCUUACAGUGCAGGUAG |
|  |  | **hsa-miR-195-5p** | MS00003703  Mature microRNA | 5'UAGCAGCACAGAAAUAUUGGC |
| Menin\| (MEN1) | **Tumor suppressor gene** | **hsa-miR-24-3p** | MS00006552  Mature microRNA | 5'UGGCUCAGUUCAGCAGGAACAG |
|  |  | **hsa-miR-29b-3p** | MS00006566  Mature microRNA | 5'UAGCACCAUUUGAAAUCAGUGUU |
| Calcium sensing receptor (CASR) | **Tumor suppressor gene** | **hsa-miR-31-5p** | MS00003290  Mature microRNA | 5'AGGCAAGAUGCUGGCAUAGCU |
|  |  | **hsa-miR-135b-5p** | MS00003472  Mature microRNA | 5'UAUGGCUUUUCAUUCCUAUGUGA |
| Catenin beta-1 (CTNNB1) | **Oncogene** | **hsa-miR-330-3p** | MS00031738  Mature microRNA | 5'GCAAAGCACACGGCCUGCAGAGA |
|  |  | **hsa-miR-483-3p** | MS00009751  Mature microRNA | 5'UCACUCCUCUCCUCCCGUCUU |
| Cyclin-dependent kinase inhibitor (CDKN1B) | **Tumor suppressor gene** | **hsa-miR-186-5p** | MS00003654  Mature microRNA | 5'CAAAGAAUUCUCCUUUUGGGCU |
|  |  | **hsa-miR-877-5p** | MS00032123  Mature microRNA | 5’GUAGAGGAGAUGGCGCAGGG |
|  |  |  |  |  |

hsa, * **Conserved among mammals in the most prevalent transcript**

has, human sapiens; miR, microRNA
